# Effect of oral beta-blocker on short and long-term mortality in patients with acute respiratory failure: results from the BASEL-II-ICU study

**DOI:** 10.1186/cc9317

**Published:** 2010-11-03

**Authors:** Markus Noveanu, Tobias Breidthardt, Tobias Reichlin, Etienne Gayat, Mihael Potocki, Hans Pargger, Antje Heise, Julia Meissner, Raphael Twerenbold, Natalia Muravitskaya, Alexandre Mebazaa, Christian Mueller

**Affiliations:** 1 Department of Internal Medicine, University Hospital Basel, Petersgraben 4, 4053 Basel, Switzerland; 2 Department of Cardiology, University Hospital Basel, Petersgraben 4, 4053 Basel, Switzerland; 3 Department of Anesthesiology and Critical Care Medicine, Université Paris Diderot and Hospital Lariboisière, 2, rue Ambroise - Paré, 75475 PARIS Cedex 10, France; 4 Operative Intensive Care, University Hospital Basel, Petersgraben 4, 4053 Basel, Switzerland; 5 Intensive Care Unit, Spital Thun-Simmental AG, Krankenhausstrasse 12, 3600 Thun, Switzerland

## Abstract

**Introduction:**

Acute respiratory failure (ARF) is responsible for about one-third of intensive care unit (ICU) admissions and is associated with adverse outcomes. Predictors of short- and long-term outcomes in unselected ICU-patients with ARF are ill-defined. The purpose of this analysis was to determine predictors of in-hospital and one-year mortality and assess the effects of oral beta-blockers in unselected ICU patients with ARF included in the BASEL-II-ICU study.

**Methods:**

The BASEL II-ICU study was a prospective, multicenter, randomized, single-blinded, controlled trial of 314 (mean age 70 (62 to 79) years) ICU patients with ARF evaluating impact of a B-type natriuretic peptide- (BNP) guided management strategy on short-term outcomes.

**Results:**

In-hospital mortality was 16% (51 patients) and one-year mortality 41% (128 patients). Multivariate analysis assessed that oral beta-blockers at admission were associated with a lower risk of both in-hospital (HR 0.33 (0.14 to 0.74) *P *= 0.007) and one-year mortality (HR 0.29 (0.16 to 0.51) *P *= 0.0003). Kaplan-Meier analysis confirmed the lower mortality in ARF patients when admitted with oral beta-blocker and further shows that the beneficial effect of oral beta-blockers at admission holds true in the two subgroups of patients with ARF related to cardiac or non-cardiac causes. Kaplan-Meier analysis also shows that administration of oral beta-blockers before hospital discharge gives striking additional beneficial effects on one-year mortality.

**Conclusions:**

Established beta-blocker therapy appears to be associated with a reduced mortality in ICU patients with acute respiratory failure. Cessation of established therapy appears to be hazardous. Initiation of therapy prior to discharge appears to confer benefit. This finding was seen regardless of the cardiac or non-cardiac etiology of respiratory failure.

**Trial registration:**

clinicalTrials.gov Identifier: NCT00130559

## Introduction

Acute respiratory failure (ARF) is responsible for about 30% of intensive care unit (ICU) admissions and is a major complication in patients already treated in the ICU [1-3]. This serious condition was shown to be associated with high morbidity and mortality rates [1-4]. Acute decompensated heart failure (ADHF), community acquired pneumonia (CAP), acute exacerbation of chronic obstructive pulmonary disease (AECOPD), pulmonary embolism (PE) and asthma are responsible for the vast majority of ICU hospitalization due to respiratory failure [5]. In-hospital mortality in ICU patients with respiratory failure is more than twice the mortality related to other ICU admissions [3].

Although mortality rates have been described in specific patient groups admitted for heart failure [6-8], severe AECOPD [9-11] or severe CAP [12-14], data concerning mortality rates and predictors of outcome in ICU patients with acute respiratory failure regardless of causal etiology are scarce. This is important for the reason that respiratory failure in one-third of ICU patients is multi-causal [15].

Accordingly, the aim of the present study was to assess in-hospital and one-year mortality in a cohort of consecutive ICU patients with acute respiratory failure indifferent of underlying etiology. We specifically determined the independent predictors of in-hospital and one-year mortality and assessed the impact of beta-blocker at admission and/or at discharge on outcome.

## Materials and methods

### Setting and study population

This report is a sub-study of the B-type natriuretic peptide (BNP) for Acute Shortness of Breath Evaluation (BASEL) II-ICU trial [15]. The goal of the BASEL II-ICU trial was to evaluate impact of a BNP-guided management strategy on outcome (hospital length of stay and costs) in ICU patients with acute respiratory failure. The BASEL II-ICU trial was a prospective, randomized, controlled, single-blinded multicenter study. Patients were enrolled in seven ICUs (one medical and one surgical ICU of a primary care facility and five interdisciplinary ICUs of tertiary referral hospitals) in Switzerland from December 2004 to March 2007. The study was carried out according to the principles of the Declaration of Helsinki and approved by the ethical committee responsible for each hospital. Written informed consent was obtained from patients or their surrogate. Details regarding study design has been published elsewhere [15]. In brief, patients presenting with acute respiratory failure severe enough to require ICU monitoring and treatment were randomized into one of two different diagnostic strategy groups. One of these groups included admission BNP value in addition to standard diagnostic workup (BNP group), while the other group did not have BNP values (control group).

Important exclusion criteria of the BASEL II-ICU trial were an obvious trauma, a BNP measurement within the preceding six hours, severe renal disease (serum creatinine >250 μmol/L), more than 12 hours since the eligibility criteria in the ICU were met, sepsis, cardiopulmonary resuscitation within 12 hours or shock.

The adjudicated diagnosis, used in the present study, was performed by two ICU specialists on the basis of all available medical records, the response to therapy and autopsy results in those patients who died in the hospital. Adjudicated diagnosis was performed by choosing one or more diagnoses from a pre-specified list that included the following items: HF, pneumonia, AECOPD/Asthma, pulmonary embolism (PE), atelectasis, mechanical airway obstruction, pneumothorax, other or unknown. The study protocol of the BASEL II-ICU study had no influence on mechanical ventilation or non-invasive ventilation (NIV) therapy. The decision about medical treatment including NIV or mechanical intubation was made solely by the ICU staff in charge following the current guidelines of the respective hospital.

The study included 314 ICU patients with acute respiratory failure. A one-year follow-up, assessed by telephone interview of the patients, their family or the referring physician, was completed in 311 (99.3%) of patients representing our study population.

### Statistical analysis

The statistical analyses were performed with the use of the SPSS/PC software package (version 15.0, SPSS Inc., Chicago, IL, USA). Comparisons were made using the t-test, Mann-Whitney U test, Fisher's exact test and chi-square test as appropriate. Mortality risk was estimated using the Kaplan-Meier method. All prognostic relevant characteristics were identified using univariate Cox-regression analysis. The model for in-hospital mortality included the following characteristics: age, systolic and diastolic blood pressure, heart rate, breathing frequency, Glasgow coma scale, body temperature, body mass index (BMI), history of malignancy, history of congestive heart failure (CHF), history of coronary artery disease (CAD), left ventricular ejection fraction, atrial fibrillation, admission pH, HCO3, base excess, PO2/FiO2 ratio, sodium, potassium, C-reactive protein, hemoglobin, white blood count (WBC), partial thromboplastin time (PTT), creatinine, blood urea nitrogen (BUN) and uric acid levels, need for mechanical intubation, need for non-invasive ventilation, need for catecholamine and admission medical treatment (diuretics, nitrates, angiotensin converting enzyme inhibitor (ACEi)/angiotensin receptor blocker (ARB), beta-blockers, statins, aspirin (ASS)/clopidogrel, calcium antagonists, coumarines, beta-mimetics, steroids). For the one-year mortality model, discharge medication was added to all variables included in the in-hospital mortality model. All variables of the in-hospital and one-year mortality model with a univariate *P*-value ≤ 0.05 were each included in the two multivariate Cox-proportional hazard models.

## Results

### Patient characteristics and mortality

A total of 314 ICU patients (median age 70 IQR (62 to 79) years) with acute respiratory failure were analyzed in the present study. Patient characteristics are displayed in Table 1. Final discharge diagnoses are displayed in Table 2. ICU median (range) length of stay (LOS) was 3 (2 to 4) days and median in-hospital LOS 14 (9 to 22) days. Overall in-hospital mortality was 16% (51 patients), 30-day mortality was 20% (61 patients) and one-year mortality was 41% (128 patients).

**Table 1:** Baseline characteristics of study population

	All studied patients (*n *= 314)	In-hospital survivors (*n *= 263)	In-hospital non-survivors (*n *= 51)	*P*-value	One-year survivors (*n *= 183)	One-year non-survivors (*n *= 128)	*P*-value
**Demography/Scores**							
Gender (male) - *n *(%))	181 (58)	147 (56)	34 (68)	0.15	100 (55)	78 (61)	0.27
Age (year)	70(62 to 78.75)	70(61 to 79)	73(63 to 76)	0.013	69(60 to 77)	74(64 to 80)	0.003
BMI^a^	25.8(22.6 to 30.8)	25.85(22.6 to 30.8)	25.8(22.5 to 28.4)	0.06	26.15(23.4 to 31.1)	25.3(21.2 to 29.1)	0.008
SAPS^b ^II score	32(26 to 46)	32(24 to 45)	36 (31 to 46)	0.12	32(24 to 45)	44(30 to 44)	0.07
**Hemodynamic parameters**							
Heart rate (bpm)	98(84 to 116)	97.5(83.75 to 115)	105(85 to 123)	0.16	98(83 to 116)	100(84 to 115.25)	0.8
Systolic blood pressure (mmHg)	127(111 to 148)	128(111 to 148)	127(111 to 139)	0.34	129.5(111 to 150)	126(112 to 143)	0.19
Diastolic blood pressure (mmHg)	67(56 to 80)	67(57 to 80)	64(53 to 78)	0.17	70(59 to 82)	62(53 to 74.5)	0.006
Mean arterial pressure (mmHg)	85(73 to 100)	87(74 to 101)	85(73 to 96)	0.15	89(76.25 to 103)	83(71.5 to 95)	0.04
Left ventricular ejection fraction (%)^c^	55(35 to 60)	50(35 to 60)	60(35 to 65)	0.35	50(35 to 60)	60(43.5 to 63.75)	0.26
Atrial fibrillation- *n* (%)	50 (16)	37 (14)	13 (26)	0.04	22 (12)	28 (22)	0.02
**Respiratory/metabolic parameters**							
Mechanical ventilation -* n* (%)	42 (13)	34 (13)	8 (16)	0.59	26 (14)	16 (13)	0.66
Non-invasive ventilation - *n* (%)	158 (50)	131 (59)	27 (53)	0.68	87 (48)	70 (55)	0.21
Breathing frequency (cpm)	24(19 to 30)	24(19 to 30)	25(20 to 30)	0.75	24(18 to 30)	25(20 to 30)	0.38
PaO2/FiO2	161(101 to 240)	169(101 to 239)	144 (92 to 216)	0.20	169 (99 to 248)	152(100 to 228)	0.41
PaCO2 (kPa)	5.9(4.9 to 7.8)	5.9(5 to 7.8)	5.8(4.8 to 8.2)	0.33	5.9(5 to 7.5)	6.05(4.8 to 8.4)	0.34
**Laboratory parameters**							
Hemoglobin (g/l)	118(101 to 141)	120(102 to 142)	108(97 to 128)	0.04	121(101 to 145)	114(100 to 134)	0.013
Uric acid (μmol/l)	381(275.5 to 502)	370(274 to 494.5)	412(311 to 521)	0.31	362(278 to 470.5)	397(273 to 557)	0.10
eGFR MDRD^d ^(mL/min/1.73 m^2^)	69(46 to 99)	71.5(46 to 102)	56(45 to 88)	0.04	72(49 to 102.75)	60.5(41.75 to 95)	0.08
Blood urea nitrogen (mg/dl)	21(13 to 33)	19(12 to 31)	26(18 to 38)	0.04	19(12 to 28)	24(14 to 39.5)	0.01
**Comorbidities to *n *(%)**							
History of heart failure	85 (27)	71 (27)	14 (28)	0.94	48 (26)	37 (29)	0.60
History of coronary artery disease	119 (38)	95 (36)	24 (47)	0.14	61 (33)	58 (45)	0.03*
Hystory of hypertension	165 (53)	114 (55)	21 (41)	0.075	99 (54)	65 (51)	0.56
Hystory of COPD^e^	123 (39)	105 (40)	18 (35)	0.53	69 (38)	54 (42)	0.42
History of malignancy	61 (19)	41 (16)	20 (39)	< 0.0001	21 (12)	39 (31)	< 0.0001
**Etiology of respiratoy failure- *n* (%)**							
Heart failure (HF) alone	101 (32)	86 (33)	15 (30)	0.64	67 (37)	34 (27)	0.06
HF + any additional diagnosis	75 (24)	61 (23)	14 (28)	0.51	40 (22)	35 (27)	0.26
HF + pneumonia	42 (14)	32 (12)	10 (20)	0.15	16 (9)	26 (20)	0.003
HF + AECOPD^f^	20 (6)	18 (7)	2 (4)	0.44	17 (9)	3 (2)	0.014
HF + other diagnosis	13 (4)	11 (4)	2 (4)	0.93	7 (3)	6 (5)	0.71
Pneumonia	50 (16)	38 (15)	12 (24)	0.11	27 (15)	22 (17)	0.57
AECOPD	30 (10)	26 (10)	4 (8)	0.66	15 (8)	15 (12)	0.31
Pneumonia + AECOPD	11 (3.5)	10 (4)	1 (2)	0.52	5 (3)	6 (5)	0.36
Pulmonary embolism	15 (5)	14 (5)	1 (2)	0.31	8 (4)	6 (5)	0.90
Unknown cause	4 (1)	4 (1.5)	0 (0)	0.38	1 (1)	2 (1)	0.37
Other cause	28 (9)	24 (9)	4 (8)	0.77	20 (11)	8 (6)	0.16
**Admission medication - *n* (%)**							
Diuretics	135 (52)	117 (53)	18 (47)	0.52	84 (56)	49 (46)	0.12
Nitrates	29 (11)	27 (12)	2 (5)	0.19	22 (15)	7 (7)	0.04
ACEi/ARB^g^	144 (46)	126 (48)	18 (35)	0.09	91 (50)	50 (39)	0.06
Beta-blocker	101 (32)	94 (36)	7 (14)	0.001	81 (44)	20 (16)	< 0.0001
Statins	80 (31)	67 (30)	13 (33)	0.69	54 (36)	26 (24)	0.05
Aspirin/Clopidogrel	102 (39)	86 (39)	16 (41)	0.80	67 (44)	35 (33)	0.07
Calcium-antagonists	43 (17)	36 (16)	7 (18)	0.79	25 (17)	18 (17)	0.92
Coumarines	86 (33)	77 (35)	9 (23)	0.15	46 (31)	39 (37)	0.31
Beta-mimetics	94 (36)	78 (35)	16 (41)	0.48	49 (33)	44 (41)	0.15
Oral steroids	45 (17)	38 (17)	7 (18)	0.89	28 (19)	16 (15)	0.45
**Discharge medication - *n *(%)**							
Diuretics	130 (41)	-	-	-	86 (47)	44 (34)	0.12
Nitrates	39 (13)	-	-	-	23 (13)	16 (13)	0.966
ACEi/ARB	160 (51)	-	-	-	121 (66)	38 (30)	0.010
Beta-blocker	119 (38)	-	-	-	84 (46)	34 (27)	< 0.001
Statins	77 (25)	-	-	-	56 (31)	20 (16)	0.001
Aspirin/Clopidogrel	91 (29)	-	-	-	61 (33)	29 (23)	0.024
Calcium-antagonists	30 (10)	-	-	-	22 (12)	8 (6)	0.057
Coumarines	104 (33)	-	-	-	67 (37)	36 (28)	0.075
Beta-mimetics	89 (28)	-	-	-	55 (30)	32 (25)	0.272
Oral steroids	41 (13)	-	-	-	20 (11)	21 (16)	0.156

^a ^BMI, body mass index (mass (kg)/height (m)^2^); ^b ^SAPS 2, Simplified Acute Physiology Score [45]; ^c ^measured by echocardiography in 128 patients; ^d ^estimated glomerular filtration rate using Modification of Diet in Renal Disease (MDRD) formula [46]; ^e ^COPD, chronic obstructive pulmonary disease; ^f ^AECOPD, acute exacerbation of COPD; ^g ^ACEi, angiotensin-converting enzyme inhibitors. ARB, angiotensin receptor blocker

Values are displayed as median (interquartile range) or number of patients (%).

**Table 2:** Final discharge diagnoses of studied patients

Characteristic	(*n *= 314)
**Heart failure (HF)**	101 (32)
**HF + any additional diagnosis**	75 (24)
**HF + pneumonia**	42 (13)
**HF + obstructive pulmonary disease**	20 (6)
**HF + other diagnosis**	13 (4)
**Pneumonia**	50 (16)
**Obstructive pulmonary disease**	30 (10)
**Pneumonia + obstructive pulmonary disease**	11 (3)
**Pulmonary embolism**	15 (5)
**Unknown cause**	4 (1)
**Other cause^a^**	28 (9)

^a^Including aspiration, anaemia, atelectasis, pneumothorax, oversedation, interstitial lung disease, obesity hypoventilation syndrome and pleural effusion.

Values are displayed as number of patients (%).

### Risk factors of one-year and in-hospital mortality

Univariate analysis demonstrates that age, a history of CAD or malignancy, BMI, diastolic blood pressure, atrial fibrillation, creatinine, blood urea nitrogen (BUN) or uric acid levels as well as treatment with oral steroids at discharge were associated with an increased risk of one-year mortality (Table 3). By contrast, treatment with oral beta-blockers, statins, aspirin and/or clopidogrel at admission, as well as ACEi/ARB at discharge was associated with a lower risk for one-year mortality. Multivariate analysis shows that history of CAD or history of malignancy was associated with an increased risk and oral beta-blocker treatment prior to admission with a decreased risk of one-year mortality (Table 4).

**Table 3:** Predictors of one-year mortality by univariate analysis (*n *= 314)

	HR (95%CI)	*P*-value
**Age**	1,03 (1.01 to 1.06)	0.0012
**Diastolic blood pressure**	0.98 (0.97 to 0.99)	0.0025
**Body mass index**	0.96 (0.92 to 0.98)	0.031
**History of malignancy**	1.99 (1.18 to 3.32)	0.0093
**Atrial fibrillation**	1.86 (1.06 to 3.33)	0.033
**Creatinin levels at admission**	1.00 (1 to 1.01)	0.048
**Blood urea nitrogen levels at admission**	1.01 (1 to 1.02)	0.02
**Uric acid levels at admission**	1.00 (1 to 1)	0.048
**Beta-blockers at admission**	0.32 (0.18 to 0.52)	<0.0001
**Statins at admission**	0.51 (0.28 to 0.94)	0.03
**Aspirin/Clopidogrel at admission**	0.56 (0.33 to 0.95)	0.03
**ACEi/ARB at discharge**	0.56 (0.36 to 0.88)	0.011
**Oral steroids at discharge**	2.34 (1.37 to 4.01)	0.0019

ACEi, angiotensin-converting enzyme inhibitors; ARB, angiotensin receptor blocker; CI, confidence interval; HR, hazard ratio.

**Table 4:** Independent predictors of in-hospital and one-year mortality by multivariate analysis

	In-hospital mortality (*n *= 51)		One-year overall mortality (*n *= 128)	
	HR (95% CI)	*P*-value	HR (95% CI)	*P*-value
**Beta-blockers at admission**	0.33 (0.14 to 0.74)	0.007	0.29 (0.16 to 0.51)	0.0003
**History of malignancy**	2.7 (1.5 to 4.9)	0.0012	2.75 (1.70 to 4.43)	0.0003
**History of coronary artery disease**	-	-	1.81 (1.15 to 2.82)	0.009

CI, indicates confidence interval; HR, hazard ratio.

Univariate analysis shows that a history of malignancy, BMI, atrial fibrillation and creatinine levels on admission were associated with an increased risk of in-hospital mortality. By contrast, treatment with oral beta-blockers prior to admission was associated with a lower risk of in-hospital mortality. Multivariate analysis shows that history of malignancy was associated with an increased risk and oral beta-blocker treatment prior to admission with a decreased risk of in-hospital mortality in ICU patients with acute respiratory failure (Table 4).

### Impact of oral beta-blockers on short and long term outcome

**Figure 1:**
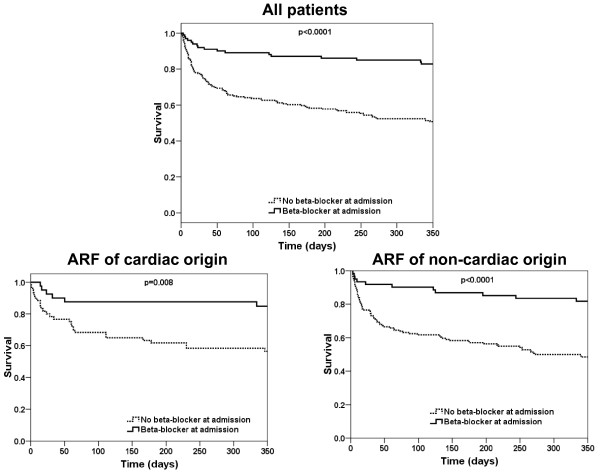
**Impact of beta-blocker at admission on long-term outcome**. Upper panel: Kaplan-Meier curve displaying overall one-year mortality in ICU patients with acute respiratory failure with or without treatment with beta-blocker at admission (*P *< 0.001 by Log Rank). Lower panel: Kaplan-Meier curve displaying one-year mortality with or without treatment with beta-blocker at admission in patients with cardiac aetiology of respiratory failure (adjudicated final diagnosis of heart failure; *P *= 0.008) and patients with non-cardiac aetiology of respiratory failure (adjudicated final diagnosis other than heart failure; *P *< 0.0001).

Table 5 displays the different beta-blocker agents and the mean dosage administered during hospitalization. Kaplan-Meier analysis confirmed a lower in-hospital and one-year mortality in ARF patients admitted with than without oral beta-blockers (*P *= 0.001 for in-hospital and *P *< 0.001 for one-year mortality respectively) (Figure 1). The beneficial effect of oral beta-blockers at admission on one-year mortality holds true in the two subgroups of ARF related to cardiac or non-cardiac causes (Figure 1).

**Table 5:** Different agents and mean dosages of beta-blocker administered at presentation, at 24 hours and at discharge

Beta-blocker	Hospital admission n (%)	mean dosage (mg)	24-hour n (%)	mean dosage (mg)	Hospital discharge n (%)	mean dosage (mg)
**Metoprolol**	36 (36)	100 (50 to 125)	30 (36)	100 (50 to 125	51 (43)	100 (50 to 125
**Carvedilol**	18 (18)	12.5 (6.25 to 25)	16 (19)	12.5 (6.25 to 25)	20 (17)	12.5 (7.81 to 25)
**Bisoprolol**	16 (16)	5 (5 to 8.75)	13 (16)	5 (5 to 5)	19 (16)	5 (5 to 5)
**Nebivolol**	22 (22)	5 (3.75 to 7.5)	19 (24)	5 (2.5 to 7.5)	26 (22)	5 (2.5 to 7.5)
**Atenolol**	4 (4)	62 (50 to 94)	2 (2.5)	75 (50 to 100)	1 (1)	100
**Sotalol**	3 (3)	160	0	-	0	-
**Celiproplol**	2 (2)	200	2 (2.5)	150 (100 to 200)	1 (1)	200

Values are displayed as number of patients (%) and mean (quartiles) dosage in mg.

**Figure 2:**
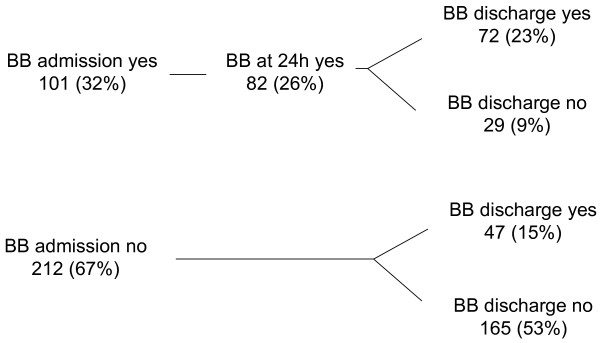
**Progress of beta-blocker therapy during course of hospitalization**. (admission, 24 hours and discharge *n *= 313).

**Figure 3:**
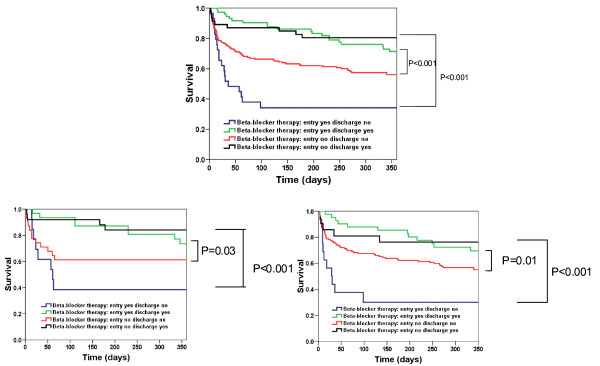
**Kaplan-Meier curve displaying mortality in patients with acute respiratory failure stratified by treatment with beta-blocker**. Left Kaplan-Meier curve displaying overall long term mortality in all studied patients; middle: patients with cardiac aetiology of respiratory failure (adjudicated final diagnosis of heart failure); right: patients with non-cardiac etiology of respiratory failure.

We further explored whether oral beta-blockers at discharge would give an additional beneficial effect on long term outcome. Kaplan-Meier analysis shows that administration of oral beta-blockers before hospital discharge gives striking additional beneficial effects on one-year mortality in our ARF patients. A beneficial effect of oral beta-blockers at discharge is seen regardless of the cardiac or non-cardiac origin of ARF (Figures 2 and 3).

## Discussion

The present study focuses on the predictors of in-hospital and one-year mortality in ICU patients with acute respiratory failure. Our study confirms the negative impact of renal dysfunction on in-hospital survival and of malignancy and history of CAD on one-year survival. Further, a positive impact on one-year overall survival was seen in patients given beta-blockers prior to admission. Discontinuation of beta-blocker therapy in patients admitted on beta-blockers was associated with higher mortality.

Short and long-term mortality has been studied in some surveys and trials involving ICU patients with a primary diagnosis of ADHF, AECOPD or acute pneumonia [6-9,11-13,16]. However, data describing mortality in ICU patients admitted for acute respiratory failure indifferent to underlying etiology are rare. In the present study, in-hospital mortality was 16% and 30-day mortality 20%. This suggests that most of the initial deaths occurred during the initial hospitalization with only a few deaths occurring shortly after discharge. One-year mortality in our ICU patients was 41%, in line with mortality rates previously described in selected ICU patients hospitalized for ADHF [6], AECOPD [11,17] or severe pneumonia (14).

Our study shows for the first time that ICU patients with acute respiratory failure treated by oral beta-blockers prior to hospital admission experienced lower in-hospital and one-year mortality. The positive impact of being treated with oral beta-blockers at the time of respiratory failure in ICU patients was unknown. Exact mechanisms of a better short term and long-term survival in patients being treated with oral beta-blockers at the time of respiratory failure remained to be explored. One assumable explication may be the relevant co-morbidities found in our patients including history of CAD in 38%, history of CHF in 27%, arterial hypertension in 53% and COPD in 39% and the positive effect of beta-blocker therapy in these different diseases. This may include an adequate control of the sympathetic nervous system in patients with CAD, CHF or arterial hypertension as well as a possible improvement of bronchodilator responsiveness and effectiveness of inhaled β_2_-sympathicomimetics in patients with AECOPD.

More importantly, we could demonstrate that discontinuation of beta-blocker therapy during hospitalization is associated with higher mortality rates, suggesting a protective effect of beta-blocker therapy in our acute respiratory failure patients. Discontinuation of beta-blocker therapy is indeed associated with a "withdrawal syndrome", a transient sympathetic hyper-response caused by hypersensitivity of cardiac β-receptors [18]. Patients in whom beta-blockers were discontinued complained of transient palpitations, tremor, sweating, headache and general malaise. A significant increase in blood pressure and heart rate could also be demonstrated 24 h after beta-blocker withdrawal [19]. A survival benefit of continuation of beta-blocker therapy in patients with ADHF was demonstrated by Butler *et al*. [20] and recently confirmed by Fonarow *et al*. [21], Jondeau *et al*. [22] and Orso *et al*. [23]. There is, furthermore, evidence that patients admitted with AECOPD may also benefit from continuation of beta-blocker therapy [24]. The observed positive association of beta-blocker continuation with lower mortality may be explained by the prevention of malignant ventricular arrhythmias, protection against myocardial infarction or acute negative mechanical remodeling, which may initiate the development of fatal pump failure [23,25].

In our study, treatment with beta-blockers at discharge was associated with lower one-year mortality. There is solid evidence showing that oral treatment with beta-blockers improves long-term survival in various cardiovascular diseases including CHF, CAD or arterial hypertension [26-29]. A recently published, large observational cohort study demonstrated that treatment with beta-blockers also reduce risk of exacerbations and improve survival in patients with COPD [30]. Interestingly, this effect was shown to be independent of cardiovascular co-morbidities. Beta-blockers are known to temper the sympathetic nervous system, including the reduction of heart rate. Therefore, negative systemic effects in the disease progression of cardiovascular disease including CAD, CHF or arterial hypertension, as well as COPD [31] could be diminished. Heart rate reduction itself may be an important mechanism of the benefit of beta-blockers. Large epidemiological studies have shown that resting heart rate was an independent predictor of all-cause mortality in individuals with and without cardiovascular disease [24].

Angiotensin converting enzyme inhibitors (ACEi)/angiotensin receptor blockers (ARB) and beta-blockers build the mainstay of therapy in patients with CHF and/or CAD with impaired left ventricular function [32]. In our study, treatment with ACEi/ARB was also associated with improved one-year survival. Importantly, lower in-hospital and one-year mortality benefits of beta-blocker therapy demonstrated in our study was independent of concomitant ACEi/ARB treatment.

Interestingly, the present study shows that the beneficial effect of beta-blockers on survival was consistently present regardless of a cardiac or non-cardiac etiology of respiratory failure. The beneficial effect of beta-blockers in the non-cardiac respiratory failure group might seem to be a paradox. However, again the high incidence of relevant cardiovascular co-morbidities known to benefit from beta-blocker treatment may explain this finding. Beta-blocker treatment has been shown to reduce mortality in patients with COPD and arterial hypertension compared with other antihypertensive agents and to reduce cardiac toxicity of short-acting beta-agonists [33,34].

Our study corroborates and extends this finding to ICU patients with respiratory failure. While early diagnosis is often difficult to perform in ICU patients presenting with acute respiratory failure, this finding may be of major clinical importance. Roughly one-third of our patients were treated with beta-blockers at admission suggesting frequent uncertainty in ICU physicians regarding the question of whether beta-blocker therapy should be continued or not. Our data advocate for a continuation of beta-blocker therapy in this patient group, although study design and power were not conceived for analysis of this issue.

In our study elevated uric acid levels were associated with increased one-year mortality in univariate analysis. In patients admitted with acute dyspnea at the emergency department, uric acid levels were demonstrated to be higher in dyspnea due to ADHF compared to other etiologies [35]. In this study uric acid levels also independently predicted two-year all-cause mortality. Our study expands these findings to ICU patients with acute respiratory failure. Uric acid is known to be associated with most cardiovascular risk factors and components of the metabolic syndrome including arterial hypertension, hyperlipidemia, or diabetes mellitus [36-38]. Uric acid levels reflect the degree of circulating xanthine oxidase activity which is stimulated by various cardiovascular diseases and is an important source of free radicals [39,40]. Accordingly, levels of uric acid might reflect a composite of cardiovascular risk factors.

Another important predictor of one-year mortality in our study was a low BMI. Previous studies demonstrated that a low BMI is associated with adverse outcome. This finding was recently confirmed in a large ICU database including 41,011 patients [41]. In this study low BMI also prolonged ICU and hospital length of stay. These findings were regardless of severity of illness quantified by SAPS II score.

A more intriguing finding of our study was the association of a low diastolic blood pressure with increased one-year mortality, even when only found in univariate analysis. At the same time, beta-blocker treatment which lowers diastolic blood pressure improved outcome. Low diastolic blood pressure is known to affect microcirculation particularly in the coronary bed, and was previously demonstrated to be associated with higher mortality in older patients [42]. Patients with severe forms of hypertension and overt coronary ischemia especially show a J-shaped relation between diastolic blood pressure during treatment and myocardial infarction [43]. The J-curve seems to be independent of treatment, pulse pressure, and the degree of decrease in diastolic blood pressure, and is unlikely to be caused by poor left ventricular function. The most probable explanation is that subjects who have severe coronary artery disease and concomitant arterial hypertension may have a poor coronary flow reserve, which makes the myocardium vulnerable to coronary perfusion pressures that are tolerated by patients without ischemia, particularly at high heart rates [44]. The most suitable explanation for this conflicting finding in our study is that patients with acute coronary syndrome as well as patients with shock were excluded due to study protocol. Patients included in our study had diastolic blood pressures that were still in a normal range (mean 62; 95%CI (53 to 74.5) mmHg).

### Study limitations

There are limitations to our study design and conclusions, related to the *post hoc *nature of the analyses. Patients were not randomized into the study according to the beta-blocker status at baseline. However, patients currently being treated with oral beta-blockers at the time of acute respiratory failure had consistently lower in-hospital and one-year overall mortality. Accordingly, the impact of beta-blocker therapy on in-hospital and one-year survival merits further confirmation by an appropriate trial. Also, data regarding duration of beta-blocker therapy prior to admission, as well as percentage of beta-blocker therapy at one-year follow-up cannot be provided. Due to the exclusion of patients with sepsis or shock our findings cannot be generalized to these subgroups of ICU patients. No adjustment for APACHE or SAPS II score has been performed in our linear regression model. The most relevant variables of both severity scores have, however, been considered.

## Conclusions

In our analysis established beta-blocker therapy appears to be associated with reduced mortality in patients admitted to the intensive care unit with acute respiratory failure. Cessation of established therapy appears to be hazardous. Initiation of therapy prior to discharge appears to confer benefit. This finding was seen regardless of the cardiac or non-cardiac etiology of respiratory failure. This observation should be confirmed by a large study that is adequately powered.

## Key messages

• Beta-blocker therapy at admission appears to be associated with a reduced mortality in patients admitted to the intensive care unit with acute respiratory failure.

• Cessation of established beta-blocker therapy in ICU patients admitted with acute respiratory failure appears to be hazardous.

• Initiation of beta-blocker therapy prior to hospital discharge appears to confer benefits. This finding was seen regardless of the cardiac or non-cardiac etiology of respiratory failure.

## Abbreviations

ACEI: angiotensin converting enzyme inhibitor; ADHF: acute decompensated heart failure; AECOPD: acute exacerbation of chronic obstructive pulmonary disease; ARB: angiotensin receptor blocker; ARF: acute respiratory failure; ASS: aspirin; BASEL: Acute Shortness of Breath Evaluation; BMI: body mass index; BNP: B-type natriuretic peptide; BUN: blood urea nitrogen; CAD: coronary artery disease; CAP: community acquired pneumonia; CHF: congestive heart failure; PE: pulmonary embolism; PTT: partial thromboplastin time; WBC: white blood count.

## Competing interests

Dr. Mueller reported receiving research support from the Swiss National Science Foundation (PP00B-102853), the Swiss Heart Foundation, the Novartis Foundation, the Krokus Foundation, Abbott, Astra Zeneca, Biosite, Brahms, Roche, Siemens, and the Department of Internal Medicine, University Hospital Basel, as well as speaker honoraria from Abbott, Biosite, Brahms, Roche, and Siemens. The other authors reported no financial disclosures.

## Authors' contributions

MN made substantial contributions to conception and design, acquisition of data, analysis and interpretation of data, and the manuscript draft. TB, TR, MP, HP, AH, JM, RT, NM and AM contributed to acquisition of data and critical revision of the manuscript. AM, also, contributed to analysis and interpretation of the data and to the manuscript draft. EG contributed to analysis and interpretation of the data, critical revision of the manuscript, and important statistical support. CM contributed to conception and design, analysis and interpretation of data, manuscript draft, and critical revision of the manuscript.
